# Spontaneous Rupture of Solid Pseudopapillary Neoplasm of the Pancreas: A Case Report and Literature Review

**DOI:** 10.70352/scrj.cr.25-0079

**Published:** 2025-06-14

**Authors:** Yuta Nakaguchi, Shinji Kishi, Naoto Shirakami, Takashi Ito, Takamasa Ohnishi

**Affiliations:** 1 Department of Surgery, Nishiwaki Municipal Hospital, Nishiwaki, Hyogo, Japan; 2 Diagnostic Pathology, Nishiwaki Municipal Hospital, Nishiwaki, Hyogo, Japan

**Keywords:** spontaneous rupture, solid pseudopapillary neoplasm, pancreatic tumor

## Abstract

**INTRODUCTION:**

Pancreatic solid pseudopapillary neoplasms (SPNs) are rare tumors, accounting for 1%–3% of all pancreatic tumors, with a predilection for young women. Owing to their often asymptomatic nature, SPNs are typically discovered incidentally. Spontaneous rupture of SPNs is extremely rare, with few reported cases. Herein, we report a case of spontaneous SPN rupture and review the literature on similar cases.

**CASE PRESENTATION:**

A 17-year-old girl presented with sudden, severe left upper abdominal pain and hemorrhagic shock. Contrast-enhanced computed tomography revealed a 13-cm heterogeneous pancreatic tail tumor with internal extravasation and massive ascites, indicative of intraperitoneal hemorrhage. The patient underwent distal pancreatectomy and splenectomy. Histopathological examination confirmed SPN with no vascular invasion. Immunohistochemistry was positive for β-catenin, CD10, CD56, and synaptophysin, with a low Ki-67 index (1%–2%). The patient had an uneventful recovery and was discharged on postoperative day 13.

**CONCLUSIONS:**

Spontaneous SPN rupture is an exceedingly rare occurrence, and its underlying mechanisms remain unclear. Ruptured SPNs may pose a higher risk of recurrence and peritoneal dissemination, necessitating long-term follow-up. Further studies are needed to elucidate the factors influencing SPN rupture and its long-term implications.

## Abbreviation

SPN
: solid pseudopapillary neoplasm

## INTRODUCTION

Pancreatic SPNs were 1st described by Frantz^1)^ in 1959 and officially recognized by the World Health Organization in 1996. The origin of SPN tumors remains unclear, and they are relatively rare, accounting for only 1%–3% of all pancreatic tumors.^2)^ These tumors predominantly affect young women.^3)^ Because SPNs often present without specific symptoms, they are typically discovered incidentally. Spontaneous rupture of SPNs is extremely rare. Herein, we present a case of SPN along with a review of the relevant literature.

## CASE PRESENTATION

**Fig. 1:**
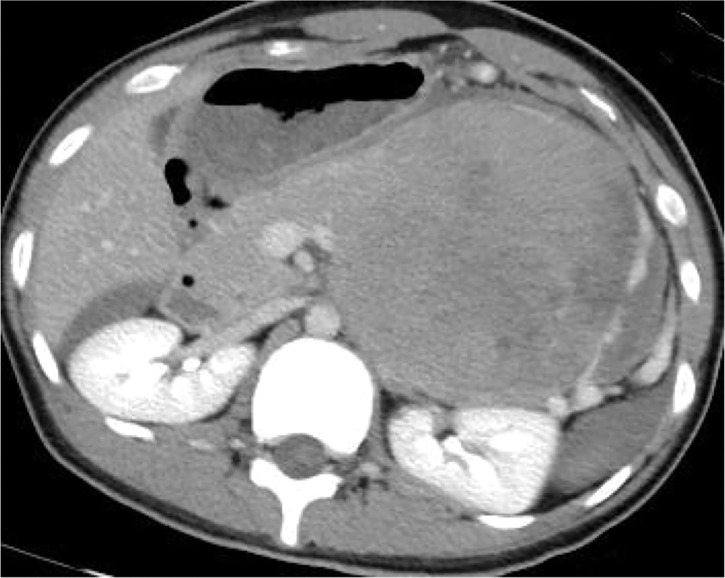
CECT reveals a well-defined 13-cm tumor contiguous with the pancreatic tail. CECT, contrast-enhanced computed tomography

**Fig. 2:**
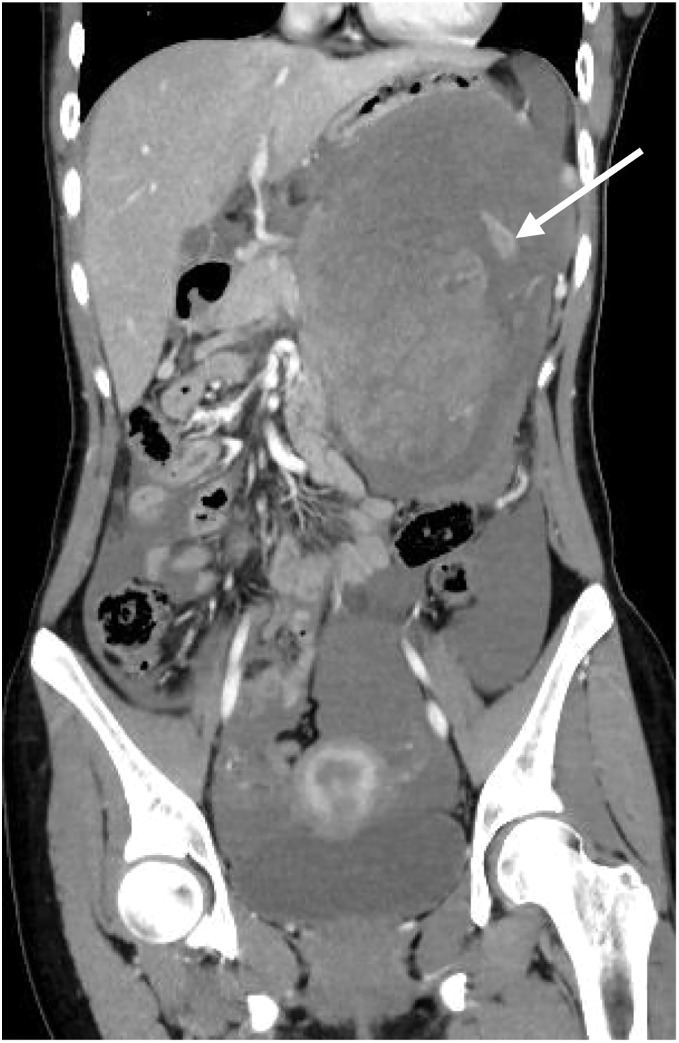
The tumor is heterogeneous and shows extravasation inside it (white arrow). CT shows massive ascites, suggesting intraperitoneal hemorrhage.

A 17-year-old girl suddenly developed severe left upper abdominal pain during class. She immediately went into shock and was rushed to the emergency department. Upon arrival at our hospital, she appeared pale, and her abdomen was distended. Abdominal examination revealed a board-like abdomen with rebound tenderness. Contrast-enhanced CT showed a well-defined 13-cm tumor contiguous with the pancreatic tail (**Fig. 1**). The tumor appeared heterogeneous with internal extravasation. Additionally, CT revealed massive ascites, indicating an intraperitoneal hemorrhage (**Fig. 2**). Based on these findings, the patient was diagnosed with an intraperitoneal hemorrhage due to the rupture of a pancreatic tumor. She subsequently underwent emergency surgery.

**Fig. 3:**
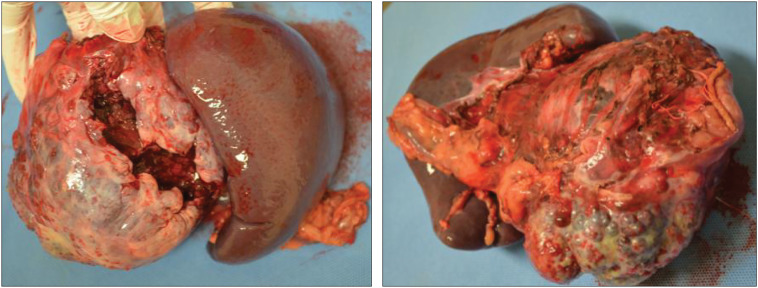
The tumor has an uneven surface and an unclear border with normal tissue. There is a 5-cm rupture of the tumor.

**Fig. 4:**
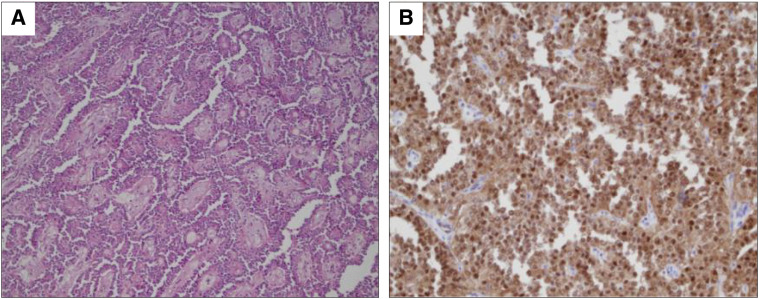
**(A)** Histologically, the tumor has small, uniform neoplastic epithelial cells growing with papillary structures and myxedematous stroma (hematoxylin and eosin; ×400). **(B)** Immunohistochemical expression of β-catenin (×400).

More than 3000 mL of bloody ascites had accumulated in the intraperitoneal cavity. After suctioning the ascitic fluid, we identified a large tumor originating from the pancreatic tail. The tumor had an uneven surface and poorly defined borders from the surrounding normal tissue. A 5-cm rupture was observed (**Fig. 3**), with persistent arterial bleeding inside the tumor. Additionally, tumor fragments were scattered throughout the abdominal cavity. These fragments were collected, and the abdominal cavity was thoroughly washed with saline. Distal pancreatectomy and splenectomy were performed, with an operative duration of 199 min. Blood loss during the surgery was 5350 mL, and the patient received a red blood cell transfusion of 3160 mL. She was discharged on postoperative day 13. Histological examination revealed that the tumor consisted of small, uniform neoplastic epithelial cells with papillary structures and myxedematous stroma (**Fig. 4**). No vascular invasion was observed. Immunohistochemistry showed positive results for β-catenin, CD10, CD56, and synaptophysin, while chromogranin A was negative. The Ki-67 labeling index was 1%–2%. The patient was ultimately diagnosed with an SPN.

## DISCUSSION

Spontaneous rupture of SPNs is rare. We identified 17 reported cases, including the present case, of spontaneous SPN rupture (**Table 1**).^4–19)^ All patients were female, with a mean age of 24 years (range: 8–51 years) and a mean tumor size of 10.9 cm (range: 5–17 cm). The primary symptoms included abdominal pain and intraperitoneal hemorrhage in 14 cases where the SPN was located in the pancreatic body or tail, and abdominal pain with upper gastrointestinal bleeding in 3 cases where the SPN was located in the pancreatic head. All patients underwent surgery, which included 13 distal pancreatectomies, 1 enucleation, and 3 pancreatoduodenectomies. Of the 17 cases, 13 required emergency surgery for reasons such as bleeding control. In 3 cases of pancreatic head tumors, hemostatic treatment was achieved via upper gastrointestinal endoscopy, allowing for elective surgery. One case of a pancreatic tail tumor was treated conservatively for retroperitoneal hemorrhage, followed by elective surgery.

**Table 1:** Reported cases of SPN with spontaneous rupture

No.	Author	Year	Sex	Age (years)	Clinical presentation	Location in pancreas	Size (cm)	Surgical procedure	Follow-up (years)	Recurrence
1	Bombí et al.^4)^	1984	Female	22	Pain, hemoperitoneum	Body	12	Emergency, DP	2	No
2	Todani et al.^5)^	1988	Female	16	Pain, hemoperitoneum	Tail	9	Emergency, DP	5	No
3	Hernandez et al.^6)^	1989	Female	22	Pain, hemoperitoneum	Tail	16	Emergency, DP	1	No
4	Benjamin Jeng et al.^7)^	1993	Female	26	Pain, hemoperitoneum	Body	13	Emergency, DP	5.5	No
5	Panieri et al.^8)^	1998	Female	34	Pain, hemoperitoneum	Body	12	Emergency, DP	Dead	NA
6	Omori et al.^9)^	2005	Female	31	Pain, hemoperitoneum	Body	10	Emergency, DP	3	No
7	Kyokane et al.^10)^	2008	Female	51	Pain, hemoperitoneum	Body/tail	11	Emergency, DP	8	6.5 years, Pe
8	Takamatsu et al.^11)^	2013	Female	13	Pain, retroperitoneal hemorrhage	Tail	5	Elective, EN	2	No
9	Huang et al.^12)^	2013	Female	29	Pain, hemoperitoneum	Body	17	Emergency, DP	1	No
10	Pattanshetti et al.^13)^	2014	Female	12	Pain, hemoperitoneum	Body	13	Emergency, DP	NA	NA
11	Rampersad et al.^14)^	2018	Female	8	Pain, hemoperitoneum	Tail	7	Emergency, DP	3	No
12	Natsume et al.^15)^	2018	Female	22	Pain	Head	8	Elective, PD	2	No
13	Nambada et al.^16)^	2019	Female	13	Pain, hemoperitoneum	Tail	NA	Emergency, DP	1.5	No
14	Xu et al.^17)^	2019	Female	22	Pain, hemoperitoneum	Body	8	Emergency, DP	1	No
15	da Silva et al.^18)^	2022	Female	31	Pain, melena	Head	12	Elective, PD	1.5	No
16	Revoredo et al.^19)^	2022	Female	44	Pain, melena	Head	6	Elective, PD	2	No
17	Our case	2022	Female	16	Pain, hemoperitoneum	Body/tail	15	Emergency, DP		

DP, distal pancreatectomy; EN, enucleation; NA, not available; PD, pancreatoduodenectomy; Pe, peritoneum; SPN, solid pseudopapillary neoplasm

In contrast, 12 cases of SPN rupture due to trauma were identified (**Table 2**).^20–31)^ This group included 4 males and 8 females, with a mean age of 14 (range: 9–23) years, which is younger than the patients with spontaneous rupture. The mean tumor size was 10 (range: 5–15) cm, similar to that of the spontaneous rupture cases. As with the spontaneous rupture cases, elective surgery was possible in the 3 cases without intra-abdominal hemorrhage. The pathogenesis of spontaneous SPN rupture remains poorly understood. SPNs are initially solid tumors but often develop a cystic component due to necrosis or hemorrhage. Generally, tumor rupture is thought to result from trauma, infection, hemorrhage, malignant transformation, or increased internal pressure. SPNs are hypervascular tumors, making them prone to hemorrhagic events. Takamatsu et al.^11)^ reported that SPNs have a natural tendency to cause hemorrhage because their cystic components arise from degeneration following intramural bleeding. Other authors who have reported cases of spontaneous rupture often share a similar view regarding the cause of rupture. Conversely, tumor rupture due to trauma is thought to be caused by external pressure that leads to bleeding and increased internal pressure. In the present case, no vascular invasion was observed, and the exact cause of spontaneous rupture remained unclear. We speculated that elevated internal pressure due to hemorrhage induced the spontaneous rupture.

**Table 2:** Reported cases of ruptured SPNs due to trauma

No.	Author	Year	Sex	Age (years)	Clinical presentation	Location in pancreas	Size (cm)	Surgical procedure	Follow-up (years)	Recurrence
1	Lieber et al.^20)^	1987	Female	13	Pain, hemoperitoneum	Body	9	Emergency, DP	2	No
2	Sanchez et al.^21)^	1990	Female	13	Pain, hemoperitoneum	Tail	10	Emergency, DP	4	No
3	PotrČ et al.^22)^	2003	Male	14	Pain, hemoperitoneum	Head	9	Emergency, PD	2.5	No
4	Kojika et al.^23)^	2004	Male	9	Pain, hemoperitoneum	Body/tail	10	Emergency, DP	NA	NA
5	Meshikes and Atassi^24)^	2004	Male	12	Pain, hemoperitoneum	Body/tail	9	Emergency, DP	3	No
6	Huang et al.^25)^	2005	Female	19	Pain, hemoperitoneum	Tail	8	Emergency, DP	0.5	No
7	Mozaffar and Abdollahi^26)^	2008	Female	14	Pain, hemoperitoneum	Body/tail	14	Emergency, DP	1	No
8	Tajima et al.^27)^	2012	Female	12	Pain	Head	14	1st: Bleeding control 2nd: PD	7	7 years, Pe
9	Park et al.^28)^	2014	Female	12	NA	Body	15	Elective, DP	8	LM
10	Mirapoğlu et al.^29)^	2016	Female	9	Pain, hemoperitoneum	Body/tail	12	Emergency, DP	1	No
11	Sano et al.^30)^	2017	Female	23	Pain, retroperitoneal hemorrhage	Body/tail	10	Elective, DP	0.75	No
12	Shimada et al.^31)^	2020	Male	16	Pain, Retroperitoneal hemorrhage	Head	5	Elective, PD	NA	NA

DP, distal pancreatectomy; LM, liver metastasis; NA, not available; PD, pancreatoduodenectomy; Pe, peritoneum; SPN, solid pseudopapillary neoplasm

The overall recurrence rate of SPNs is approximately 3%^32,33)^; however, the recurrence rate in cases of ruptured SPNs is reported to be 10%–15%.^33–36)^ Ruptures are expected to significantly impact peritoneal dissemination, although only a few cases have been reported following rupture. Due to the rarity of SPN rupture, it is unclear whether there is a correlation between rupture and peritoneal dissemination. In the previous 16 cases of spontaneously ruptured SPNs, only 1 case experienced recurrence with peritoneal dissemination 6 years and 6 months post-surgery. However, given the rarity of spontaneous SPN rupture and the short mean follow-up period of 2.75 years, it is difficult to determine whether the recurrence rate is indeed higher in these cases. Recurrence was observed in 2 of the 12 cases of traumatic rupture, but the number of cases is too small to draw definitive conclusions. However, it is suggested that the recurrence rate for both spontaneous and traumatic ruptures is higher than the general SPN recurrence rate of 3%. It is unclear whether there is a difference in recurrence rate or prognosis between different modes of rupture.

Another pancreatic tumor that can cause spontaneous rupture is mucinous cystic neoplasm (MCN). MCNs account for 1% of all pancreatic tumors and are surrounded by a thick fibrous capsule, making rupture rare. As with SPN, the pathogenesis of MCN rupture has not been elucidated, but there have been 4 cases of rupture during pregnancy, suggesting the involvement of sex hormones.^37)^ MCN is generally considered a tumor with a good prognosis, but there are reports of poor prognosis in cases of invasive carcinoma; 129 cases of mucinous cystic adenoma (MCA) diagnosed have not recurred, but there are reports of recurrence in 4 of 27 cases of mucinous cystic carcinoma (MCC).^38)^ Comparison with SPN in terms of recurrence rate and prognosis is difficult due to the small number of cases.

SPNs are neoplasms with low malignant potential and tend to grow slowly. They appear to have a predilection for young women, and there have been instances of recurrence more than 20 years after surgery,^39,40)^ indicating the necessity for long-term follow-up.

## CONCLUSIONS

Spontaneous rupture of SPN is an exceptionally rare event, with limited reported cases. In this study, we presented a case of a ruptured SPN in a young female patient, highlighting the challenges in diagnosing and managing this condition. While the exact mechanism of rupture remains unclear, elevated internal pressure due to hemorrhage is a likely contributing factor. Surgical resection remains the standard treatment, with a generally favorable prognosis. However, given the potential risk of recurrence, particularly in ruptured cases, long-term follow-up is essential to monitor for peritoneal dissemination and late recurrence. Further studies are needed to better understand the pathogenesis and long-term outcomes of ruptured SPNs.

## ACKNOWLEDGMENTS

We would like to thank Editage (www.editage.jp) for English language editing.

## DECLARATIONS

### Funding

No funding was involved in this case report.

### Authors’ contributions

YN wrote the initial draft of the paper.

SK, NS, TI, and TO supervised this study and revised the paper.

All authors read and approved the final manuscript.

All authors consent to take responsibility for all aspects of the research.

### Availability of data and materials

The data that support the findings of this study are not available for public access due to patient privacy concerns, but are available upon reasonable request from the corresponding author, Yuta Nakaguchi.

### Ethics approval and consent to participate

This work does not require ethical considerations or approval. Informed consent to participate in this study was obtained from the patient.

### Consent for publication

The case report and publication process were explained to the patient, who granted permission to publish the report.

### Competing interests

The authors declare that they have no competing interests.
